# Epidemiology of Thyroid Cancer in an Area of Epidemic Thyroid Goiter

**DOI:** 10.1155/2013/584768

**Published:** 2013-03-04

**Authors:** Antonio Cossu, Mario Budroni, Panagiotis Paliogiannis, Giuseppe Palmieri, Fabrizio Scognamillo, Rosaria Cesaraccio, Federico Attene, Mario Trignano, Francesco Tanda

**Affiliations:** ^1^Department of Surgical, Microsurgical and Medical Sciences, University of Sassari, Via le San Pietro 43B, 07100 Sassari, Italy; ^2^Service of Epidemiology, ASL 1, Via Amendola 55, 07100 Sassari, Italy; ^3^Institute of Biomolecular Chemistry, Cancer Genetics Unit, CNR, Traversa La Crucca 3, 07040 Sassari, Italy

## Abstract

The aim of this study was to analyze and describe the epidemiological characteristics and trends of thyroid cancer in the province of Sassari (Sardinia, Italy), an area with epidemic thyroid goiter, in the period 1992–2010. Data were obtained from the local tumor registry which makes part of a wider registry web, coordinated today by the Italian Association for Tumor Registries. An increasing trend in the incidence of thyroid cancer in the province of Sassari was evidenced. This trend seems to follow the general worldwide trend and does not seem to be related to the high incidence of thyroid goiter in the area. The frequencies of the different histological subtypes were similar to those reported in numerous national and international reports. Women are affected earlier than men and, therefore, suffer greater professional, economic, and social impacts. Overall mortality is low and a relative 5-year survival is excellent, especially in comparison to other malignancies.

## 1. Introduction

Thyroid cancer accounts for approximately 2% of all cancers diagnosed worldwide and 95% of all endocrine cancers [1]. Recent reports describe a continuous increase in thyroid cancer incidence worldwide. In certain geographical areas, this increase exceeds 100% (and is as high as 250% in some cases) [2]. By contrast, small declines in incidence were registered in a few areas [2]. It has been suggested that this generally steady trend of increasing thyroid cancer incidence may be only apparent, and that it depends on higher detection rates of small tumors due to the technological advances in morphological and functional imaging techniques over the last few decades [3].

Thyroid goiter is a pathological condition with epidemic dimensions in some areas because of several geographical, nutritional, and genetic factors. It has been linked to a higher risk of thyroid cancer, especially the follicular histotype [4].

The aim of this population-based study was to analyze and describe the epidemiological characteristics and trends of thyroid cancer in the province of Sassari (Sardinia, Italy), an area with epidemic thyroid goiter, in the period 1992–2010.

## 2. Materials and Methods

The epidemiological data presented in this paper were obtained from the “Registry of the Tumors of the province of Sassari.” This registry was created in 1992 by the local health agency for the epidemiological surveillance of tumors in the province. In 1999, it became part of a wider web of tumor registries, coordinated today by the Italian Association for Tumor Registries (Associazione Italiana Registri Tumori (AIRTUM)). The association coordinates 34 registries in the country, collects and publishes data, and collaborates with international organizations in the field.

Every registry collects data on tumoral diseases affecting inhabitants in the territory of jurisdiction through the local hospitals and health care services, as with other registries (e.g., death registries). Demographic, clinical, pathological, and prognostic data are collected for each case of cancer and are registered in a digital database. This database was the data source for the present population-based report.

The demographic characteristics of the patients affected by thyroid cancer were collected. Crude incidence and mortality rates per 100,000 inhabitants per year were calculated, as were standardized rates adjusted for European population standards. A comparison between incidence and mortality in the province of Sassari and those in other Italian provinces was performed. Additionally, the cumulative risk of developing the disease and of dying between zero and 74 years of age was estimated. The age class distribution and time trends of incidence, mortality, mean age of disease onset, and histology were also evaluated. Finally, a relative 5-year survival was calculated.

## 3. Results

The overall number of cases of thyroid cancer registered was 1,108. Diagnosis was confirmed by histological or cytological reports in 1,089 cases (98.3%) and using other information sources (clinical reports, death certifications, etc.) in the remaining 19 cases (1.7%). Among the 1,108 individuals registered, 237 were males and 871 females, with a male-to-female ratio of 1 : 3.7. The mean age was 52.7 years for males and 50.7 years for females. The cumulative risk of developing the disease was 0.42% for males and 1.49% for females.

The crude incidence of thyroid malignancies in the period under investigation was 5.8/100,000 for men and 20.5/100,000 for women. Standardized incidence rates were 5.2/100,000 for males and 18.4/100,000 for females.

**Figure 1:**
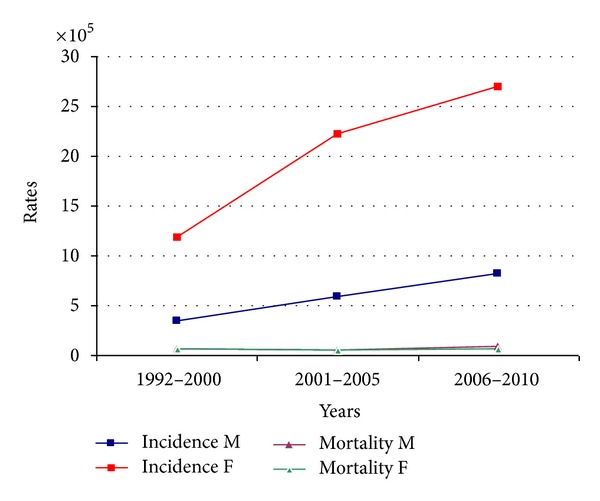
Incidence and mortality rates trends.

**Figure 2:**
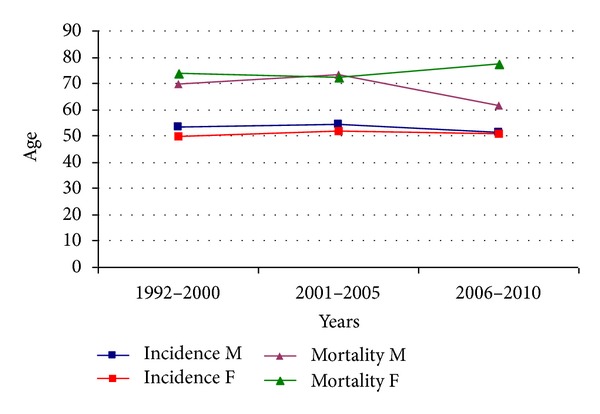
Trends of mean age at disease onset and death.

Table 1 shows the distribution of incidence in relation to age in percentages while Table 2 shows the distribution of incidence rates in relation to age. Peak incidence occurred at 80–84 years for males and at 50–54 years in females. Incidence rates were also calculated for the following three time periods: 1992–2000, 2001–2005, and 2006–2010 (Figure 1). There was a progressive increase in the incidence rate in males, from 3.37/100,000 in the first period to 5.87/100,000 in the second period and 8.13/100,000 in the last period. The corresponding figures for females were 11.85/100,000, 22.14/100,000, and 26.93/100,000, respectively. A twofold increase in incidence occurred between 1992 and 2010. Analysis of the trend of mean age at disease onset for the same periods of time did not reveal any relevant changes (Figure 2).

**Figure 3:**
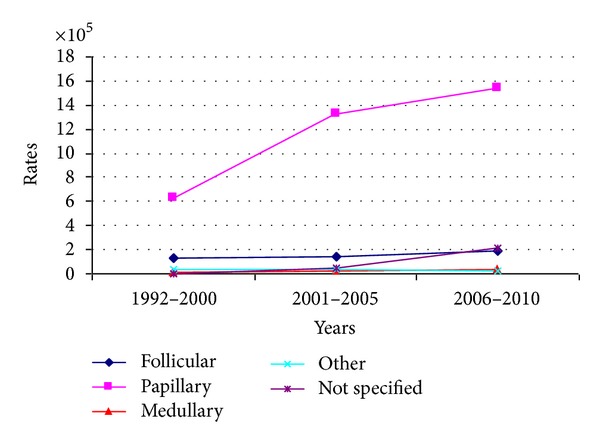
Trend of histological subtypes rates.

Concerning histology, 866 lesions (79.5%) were papillary tumors, 123 (11.3%) follicular, 20 (1.8%) medullary, and 28 (2.6%) other histological subtypes. In the remaining cases (4.8%), the exact histotype was not specified. An increasing trend with regard to the principal histotypes (papillary and follicular) was observed from 1992 to 2010, while medullary and other rare histotypes remained stable (Figure 3).

Table 3 shows the comparison of the incidence and mortality in the province of Sassari with those in other Italian provinces. There were 71 deaths in the period under investigation (32 males and 39 females). Crude overall mortality was 0.8/100,000 for males and 0.9/100,000 for females. Mean age at death was 67.9 years in males and 74.2 years in females. Standardized mortality rates were 0.8/100,000 for females and 0.6/100,000 for males. The cumulative risk of death was extremely low (0.05% for males and 0.04% for females). Table 2 shows the age-class distribution of mortality rates. There was a steady increase in mortality in relation to age after the fifth decade of life. Figure 1 shows the time trend of mortality between 1992 and 2010; there were no significant changes. Finally, a relative 1-year survival was approximately 96.5%. Relative survival at 5 years from diagnosis was 92%.

## 4. Discussion

Thyroid cancer accounts for approximately the 2% of all cancers diagnosed worldwide and 95% of all endocrine cancers [1]. Recent data suggest there were more than 213,000 new cases of thyroid cancer worldwide in 2008, with a crude incidence rate of 3.1/100,000 and a cumulative risk of developing the disease of 0.31 [5]. The male-to-female ratio was approximately 1 : 3.3, while the crude incidence for men was 1.4/100,000 and that for women was 4.9/100,000. The cumulative risk was 0.15 for males and 0.47 for females [5].

Concerning the Italian population, we obtained information using the same data source, and the following picture for 2008 was created: 7,448 new cases (1,618 males and 5,830 females), a crude incidence of 12.5/100.000 (5.6 for males and 19 for females), and a cumulative risk of 0.88 (0.4 for males and 1.35 for females). Italy ranks third in Europe in terms of new cases, after the Russian Federation and France [5].

The standardized incidence of thyroid cancer in the province of Sassari was similar to that estimated for the entire country (Table 3). This is somewhat surprising as Sardinia is an area of epidemic thyroid goiter, which is associated with increased incidence of cancer, particularly the follicular type [3]. This seems not to be the case in our province, where the percentage of follicular thyroid cancers was similar to that in large national and international reports, with only a slight tendency to increase from 1992 to 2010. Our findings are confirmed by surgical reports published by surgeons employed in local hospitals. In these reports, less than 30% of the patients who underwent thyroidectomy for thyroid disease were affected by cancer and the percentage of the follicular subtype, as determined by pathological examination of surgical specimens, was comparable to that found in the present study [6].

Comparisons of the incidence rates with those of other Italian provinces place our province approximately in the middle, between provinces with higher incidences, such as Ferrara, Modena, and Reggio Emilia, and provinces with lower incidences, such as Ragusa and Trento.

Considering the distribution of the disease in relation to age, only approximately 10% of the cases occurred in individuals younger than 30 years and older than 75. More than half of the cases were observed in individuals aged 45–74 years. It is interesting to observe that the peak incidence in men occurred at age 80–84 years, while in women it occurred earlier (at 50–54 years of age). This has relevant clinical, prognostic, and social consequences. While women generally develop the disease earlier in their lives, when they are professionally active and have a long life expectancy, men develop the disease later, probably after their retirement from work and when their life expectancy and probability of dying from the disease are lower.

The time trends analysis showed a stable increase in incidence in the province of Sassari. This trend is common in numerous national and international geographical areas, which have contributed to the global increase in the number of new thyroid cancer cases reported in several publications. As we mentioned before, in certain geographical areas such increases are greater than 100% and as high as 250% in some cases [2]. It is not clear whether this is a real increase in incidence, as was demonstrated in some areas after nuclear incidents, or whether it reflects a general improvement in the technological means and expertise employed to detect small thyroid malignancies, which in the past may have remained undiscovered [3, 7]. Several autopsy studies demonstrated a prevalence of thyroid microcarcinomas ranging from 2% to 35% [8, 9].

Concerning mortality, only 71 deaths occurred in the 18 years we studied. Crude overall mortality was 0.8/100,000 for men and slightly superior for women (0.9/100,000). These values are similar to those observed in other provinces in Italy and in recent European reports [5, 10], but somewhat higher than some worldwide estimates [5]. Considering the age-class mortality trend, a natural increase in relation to age was observed in both sexes, with peaks after the eighth decade of life and without significant variations between 1992 and 2010. The relative 5-year survival was lower in males than in females, but by global standards it can be considered excellent, especially in relation to other types of malignancies, and suggests that a good local health care system is available for the sufferers.

## 5. Conclusions

Our data show an increasing trend in the incidence of thyroid cancer in the province of Sassari. This trend seems to follow the general worldwide trend and does not seem to be related to the high incidence of thyroid goiter in the area. The frequencies of the different histological subtypes were similar to those reported in numerous national and international reports. Women are affected earlier than men and therefore suffer greater professional, economic, and social impacts. Overall mortality is low and a relative 5-year survival is excellent, especially in comparison to other malignancies.

## Figures and Tables

**Table 1:** Age-class incidence distribution.

Age (years)	Incidence (%)	
	Males	Females
0–14	0.84	0.11
15–29	10.97	9.4
30–44	20.25	26.52
45–59	30.80	32.72
60–74	26.16	24.57
75+	10.97	6.66

**Table 2:** Age-class incidence and mortality rates.

Age (years)	Incidence (/100,000 per year)		Mortality (/100,000 per year)	
	Males	Females	Males	Females
0–4	0	0	0	0
5–9	0	0	0	0
10–14	0.9	0.5	0	0
15–19	1.1	5.2	0	0
20–24	2.7	7.6	0	0
25–29	4.6	14.6	0.6	0
30–34	2.3	20	0	0
35–39	4.8	27	0	0
40–44	7.6	23	0	0
45–49	9.8	33.8	1.4	0.3
50–54	7.4	35.1	0	0
55–59	9.7	35	0.8	1.2
60–64	11.7	34.2	1.4	0.4
65–69	10.9	33.2	2.6	3.6
70–74	9.8	31	2.6	2.1
75–79	12.8	22.8	3.7	6.7
80–84	13	13.1	5.8	4.7
85+	5.9	10.8	7.9	7.6

Total	5.8	20.5	0.8	0.9

**Table 3:** Comparison with incidence and mortality rates of other Italian provinces.

Province	Incidence (/100,000 per year)		Mortality (/100,000 per year)	
	Males	Females	Males	Females
Alto Adige	3.2	6.1	0.9	1
Biella	4.2	9.5	0.7	0.6
Ferrara	8.1	24.7	0.6	0.5
Firenze	4.2	12.9	0.5	0.7
Friuli V.G.	3.6	12.8	0.6	0.9
Genova	4.5	12.9	0.5	0.5
Macerata	3.7	8	0.4	0.5
Modena	7	18.9	0.4	0.6
Napoli	4	10.8	0.9	0.7
Parma	6.1	17.1	1	0.5
Ragusa	2.8	8.7	0.3	0.8
Reggio Emilia	2.8	20.3	0.3	0.5
Romagna	5.3	18	0.4	0.7
Salerno	4.2	14.6	0.8	0.8
Sassari	**3.9**	**15**	**0.8**	**0.6**
Torino	3.1	10.4	0.3	0.7
Trento	2.9	9.4	0.4	0.7
Umbria	4.5	12.4	0.6	0.6
Varese	4.2	9.5	0.5	0.3
Veneto	3.4	10.1	0.4	0.6

MEAN	4.5	13.7	0.6	0.7
